# Antigenic Variability

**DOI:** 10.3389/fimmu.2020.02057

**Published:** 2020-09-11

**Authors:** Alexander I. Mosa

**Affiliations:** Department of Cell and Systems Biology, University of Toronto, Toronto, ON, Canada

**Keywords:** quasispecies, antibody, antigenic variability, vaccine, escape mutant

## Abstract

Protective vaccines for hypervariable pathogens are urgently needed. It has been proposed that amputating highly variable epitopes from vaccine antigens would induce the production of broadly protective antibodies targeting conserved epitopes. However, so far, these approaches have failed, partially because conserved epitopes are occluded *in vivo* and partially because co-localizing patterns of immunodominance and antigenic variability render variable epitopes the primary target for antibodies in natural infection. In this Perspective, to recast the challenge of vaccine development for hypervariable pathogens, I evaluate convergent mechanisms of adaptive variation, such as intrahost immune-mediated diversification, spatiotemporally defined antigenic space, and infection-enhancing cross-immunoreactivity. The requirements of broadly protective immune responses targeting variable pathogens are formulated in terms of cross-immunoreactivity, stoichiometric thresholds for neutralization, and the elicitation of antibodies targeting physicochemically conserved signatures within sequence variable domains.

## Introduction

**Figure 1:**
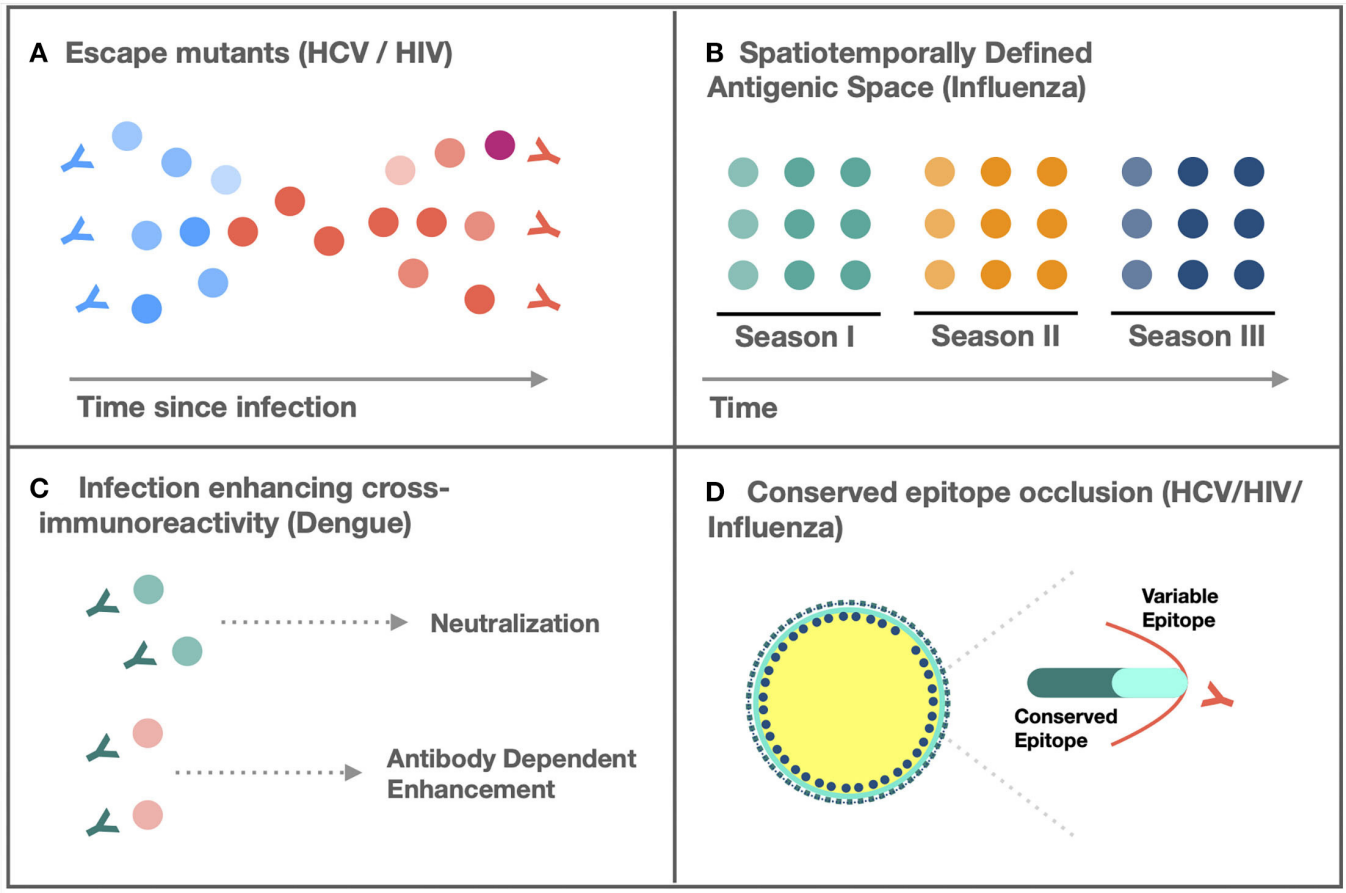
Mechanisms of adaptive variation. **(A)** Persistent generation of escape mutants resistant to neutralization by extant, intrahost antibodies prevents viral clearance (3). **(B)** Spatiotemporally defined emergence of novel antigenic variants resistant to population level immunity facilitates seasonal outbreaks (4). **(C)** Cross-immunoreactivity of antibodies can enhance infectivity of antibody bound virions (5). **(D)** Occlusion of evolutionarily constrained epitopes by variable domains limits cross-neutralization (6).

Antigenic variability is characterized by the emergence of sequence distinct variants within a species, circulating between hosts, within hosts, or temporally across populations, for which adaptive immunity elicited by one strain fails to protect against another (1). As a feature emergent from the inability of the host–immune response to match the antigenic breadth of, or enrich for constrained targets within, the infecting pathogen, sequence diversity is necessary but not sufficient for antigenic variability. Sequence variability must also be selectively preserved in epitopes targeted by the host-adaptive immune response and in which mutations confer relative resistance or sensitivity to a host-specific antibody or T-cell repertoire. The challenge of variability-mediated immune escape is exemplified by the successful introduction of vaccines for monoantigenic pathogens (small pox, measles, mumps, etc.), but the persistent difficulty in vaccine development for variable pathogens, including hepatitis C virus (HCV), human immunodeficiency virus (HIV), influenza, and dengue, which cause severe diseases characterized by hepatic cirrhosis, immunodeficiency, pneumonia, and hemorrhagic fever, respectively (2). Although mechanisms of adaptive variation differ between viruses, they converge on four patterns (Figure 1):

- Generation of an intrahost quasi-species population characterized by a mutant spectrum generated by high replicative error rates that sustain a reservoir of antigenically distinct virions with varying susceptibility to host–immune response.
- Spatiotemporally defined antigenic space leading to persistent generation of variants resistant to the regional host population's extant immunity.
- Spectra of infection-enhancing cross-immunoreactivity between quasi-species or subtypes.
- Conserved, epitope-masking by variable immunodominant epitopes, steric hindrance, or event-modulated conformational occlusion.

Neither mutually exclusive or exhaustive, these patterns typify urgent challenges in vaccine development for which traditional approaches, such as immunization by live-attenuated, subunit, or whole-particle inactivated virus, remain inadequate.

## Quasi-Species–Mediated Evasion

Quasi-species refer to individual variants in a mutant diversified population (6). Although each quasi-species is a single replicative unit, heterogenic progeny and phylogenetic convergence of contemporaneous quasi-species result in selection acting on quasi-species populations, rather than discrete variants. Phenotypes are therefore influenced by population structure, with interacting networks of co-operativity and cross-reactivity influencing the fitness of both individual variants and the population ensemble (7). Positive and negative selection forces shape the quasi-species population, with sequence-space expanded by the former, via immune-mediated diversification, and constrained by the latter, as increasing mutational load reduces mutational robustness of variants occupying distal nodes in the population network. The absolute contribution of each force to genomic structure can be proxied by ratio of synonymous to non-synonymous mutations at each codon aligned pair, with replicative functions of non-structural proteins (HCV's NS5b polymerase) preserved by strong negative selection (purging even drug-resistant variants with reduced replicative fitness), and immunodominant epitopes vulnerable to adaptive host responses diversified by the combined effects of positive selection and inherent functional plasticity (8, 9). A crucial feature of quasi-species populations is therefore selectively preserved variability in regions exposed to adaptive immune pressures, with population dynamics shaped by the fitness conferred by mutations in those residues in relation to the total mutational landscape of contemporaneous or preceding variants and their provoked immune responses.

HIV-1 and HCV, with replicative error rates of 10^−4^ and 10^−3^ per nucleotide per replication, respectively, are prototypical pathogens for studying quasi-species–mediated immune evasion (10). For both viruses, initial diversification is associated with envelope glycoprotein targeting antibodies, which alter the fitness landscape to favor minor variants in the mutant reservoir that subsequently expand to become dominant quasi-species (3). This reductionist pattern of cyclical immune escape, expansion, and clearance of dominant quasi-species has been recently questioned, with more complex dynamics involving networks of cross-reactivity possibly contributing to population dynamics (7). However, antibody-mediated positive selection of escape variants has been observed in both animal models and clinical settings, with evidence consistently implicating strain-specific neutralization by AB targeting HIV's (V1V2, C3V4) and HCV's (HVR1) variable regions (11, 12). Recent reports suggest, at least in the case of HCV, that HVR1 defined quasi-species diversity in acute infection predicts progression to chronicity, corroborating the relationship between population diversity and fitness, while suggesting antigenic variation is not only a compensatory outcome of, but preparatory mechanism to, host immune pressure (13). Unfortunately, the restricted quasi-species specificity of neutralizing antibody (nAB) responses elicited by natural infection extends to vaccination, with HIV and HCV candidates repeatedly successful in eliciting nAB responses to the homologous vaccine immunogen, but not to heterologous variants (14, 15).

## Spatiotemporally Defined Antigenic Space

Unlike quasi-species mediated evasion, in which concurrent infection with multiple variants facilitates intrahost adaptive escape, influenza exploits host-population level vulnerabilities in immune memory via sequential, seasonal generation of escape variants (4). Similar to quasi-species, however, antigenic variation between seasonal variants is enriched in immunodominant, strain-specific neutralizing epitopes (16). Subtypes stochastically generated via antigenic shift and drift are consolidated, in terms of fitness, by variations in infectiousness, tropism, and cross-immunoreactivity with extant, population-level AB. Seasonal vaccine development therefore prioritizes predicting which candidate, among previously described variants, will most effectively induce immune responses that will be cross-neutralizing to the circulating variants each year (17). The ongoing challenge in accurately predicting seasonal variants and the significant risk of emergent pandemic variants motivate development of universal vaccines (18). However, akin to HIV and HCV, immunodominance of the mutationally tolerant regions, in this case the globular head of the surface glycoprotein hemagglutinin (HA), complicates efforts (16). Encouragingly, among adults with prior infection–induced HA AB, strong recall response when challenged with novel strains, especially those sharing neutralizing epitopes, has been observed (19). This implies a partially protective, primed immunity may characterize repeated exposure to variable pathogens, whereby cross-reactive B-cell receptors (BCRs) can be stimulated by, and affinity mature to, novel strains, resulting in successively enhanced paratope affinity to cross-conserved, immunogenically subdominant epitopes.

## Cross-Reactivity, Antigenic Cooperation, and Antibody-Dependent Enhancement

Promiscuous binding of AB elicited by one antigen to another may be protective, infection-enhancing, or both, to varying extents, depending on the antigenic space. Instances of the former characterize broadly nAB (bnAB), which, via paratope interaction with conserved residues critical for viral entry, indiscriminately bind, and neutralize a population of antigenically diverse variants (20). Infection-enhancing antibodies, in contrast, target cross-reactive epitopes with varying affinities, sometimes neutralizing high-affinity variants while, through recruitment of targets of viral tropism, facilitating entry of low-affinity variants (5). Precise mechanisms notwithstanding, the varying contributions of epitope specificity, accessibility, and virion maturity in successive infection by different dengue subtypes suggest the immunological sequence following AB binding, based on Fc-mediated effector functions and paratope affinity (k**off**/k**on**) to targeted epitope(s), rather than simply the steric occupation of receptor-binding domains, mediates neutralization (5). Circumstantial contributions to antibody-mediated neutralization, among quasi-species structured viral populations, extend beyond host immune mediators to the intrahost sequence space and the corresponding network of cross-reactivity. Considered on an affinity spectrum, high-affinity variants may, via enrichment with cross-reactive AB, function as antigenic altruists by facilitating persistence of lower-affinity variants to which quasi-species–specific affinity maturation has been frustrated (7). Observations of reduced antigenic diversification and increased negative selection during chronic HCV infection, coupled with multiyear persistence of intrahost variants and quasi-species subpopulations, support a model of antigenic cooperation whereby the cross-reactive structure of the sequence space itself protects the intrahost population from AB neutralization (21).

## Conserved Epitope Masking

Conservation of physiochemical patterns, stretches of low Shannon entropy, high negative selection, and extensive convergent evolution, suggested by broad cross-reactivity, indicate that, even among antigenically variable viruses, antigenic conservation is required to preserve fitness (21, 22). The dual-selective pressures for functional conservation and immune evasion preserve a common antigenic pattern across variable viruses, marked by mutationally tolerant, immunodominant epitope(s) masking conserved, conformationally, or glycosylationally occluded, neutralizing domains (23–25). HCV, HIV, and influenza exhibit this pattern, with bnAB, already a minority of the humoral response, functionally limited by the low accessibility of targeted epitopes or the limited neutralization window afforded by receptor binding–induced conformational change (6). Ongoing research into mapping the antigenic determinants of broad neutralization and eliciting the complementary antibodies, either through reverse-vaccinology or truncation of variable epitopes from vaccine immunogens, provides a basis for rationale vaccine design (26). However, challenges in reconstituting affinity maturation through reverse vaccinology, reduced immunogenicity of variable-epitope deleted immunogens, and the occlusion of conserved epitopes *in situ* suggest effective vaccine design for antigenically variable pathogens may need to target, rather than circumvent, the hypervariable epitopes.

## Perspective

**Figure 2:**
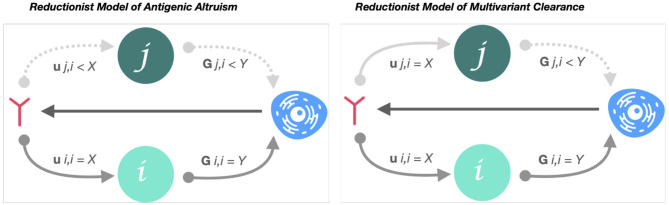
Reductionist model of low-affinity variance cross-nAB attenuation of antigenic cooperation. Reductionist model of antigenic altruism describes the probability that an immune response generated by variant *i* will be stimulated by variant *j* (G*j, i*) and the probability that an immune response to *i* neutralizes *j* (U*j, i*). Accordingly, if G*j, i* < G*i, i*, but > 0, and variant *i* preceded *j*, the response to *j* will be characterized by a variant-specific, relational immunodeficiency (antigenic cooperation) (7). However, if U*j, i* ≈ U*i, i* (low-affinity variance of nAB between *i* and *j*), variants *j* and *i* are equally vulnerable to neutralization to the immune response generated by *i*. In this case, despite *j-*specific immunodeficiency, variant *j* will be cleared with equal probability to variant *i*.

Cross-reactivity is necessary but not sufficient for a protective AB response targeting variable epitopes. To resolve infection in an antigenically convergent sequence space, cross-reactive AB must also bind neutralizing epitopes with low paratope-affinity-variance across isolates (Figure 2).

Given minor physicochemical variations can alter paratope: epitope interaction, this latter requirement may explain why persistent infection has been attributed to networks of cross-reactivity and their emergent features, such as antigenic altruism, a phenomena whereby cooperation between cross-reactive variants increases population fitness at the expense of “sacrificial” variants preferentially targeted by host immune responses (7). This requirement may also explain how, despite extensive cross-reactivity of variable epitope targeting immune responses in chronic HCV and HIV infection, neutralization-resistant escape mutants emerge: their reduced affinity to cross-reactive AB jeopardizes the stoichiometric requirements for neutralization (27). Mechanistically, this phenomenon suggests one criterion for broadly protective responses to variable pathogens is the induction of AB indiscriminately targeting physicochemically convergent signatures within sequence variable epitopes.

Evidence from both clinical cohorts and preclinical models supports the feasibility of biasing affinity maturation to conserved signatures within variable epitopes. Among patients chronically infected with HCV, length of exposure to antigenically diverse quasi-species is associated with the development of bnAB targeting constrained residues, suggesting repeated exposure to variable domains partially attenuates immunodominance and shifts affinity maturation toward better conserved, more consistently presented epitopes (28). Polyvalent malaria vaccine candidates have also been observed to increase neutralization breadth by biasing the immune response to conserved residues (29). *In silico* models, consistent with clinical findings, describe this phenomenon in terms of compromised fitness among clonal lineages with high BCR affinity to physicochemically variable, rather than conserved, residues (30). Specifically, increasing allelic inclusion in a multivalent vaccine formulation broadened cross-strain malaria neutralization by enhancing the humoral response to both conserved and polymorphic faces of malaria's apical membrane antigen 1 (30). These findings imply selection for enhanced nAB breadth following multivalent vaccination can be operative within, rather than solely between, antigenic domains and may therefore be compatible with a single-variable epitope replacing full-length antigen as the functional immunogen unit.

Indiscriminate inclusion of variants in polyvalent formulations would likely recapitulate the events in natural infection leading to strain-specific neutralization, or worse, antibody-dependent enhancement via induction of cross-reactive antibody with subneutralization threshold affinity (31). The criterion of multisubtype inclusion or sequence breadth maximization in prior multivalent HCV candidates may therefore be misguided (32). Alternatively, selecting variants based on their physicochemical, rather than sequence-specific or phylogenetic diversity, may accelerate the immune frustration postulated to favor induction of broadly reactive, low-affinity variance AB.

## Concluding Remarks

Rationale vaccine design begins with a hypothesis, informed by clinical data, animal models, and *in vitro* assays, describing a protective immune response. For hypervariable viruses, like other pathogens, these responses are multifaceted, involving coordination across innate, cellular, and humoral immunity (33). Critically, the role of a protective vaccine is not to directly stimulate each constituent of a successful immune response, but to identify, and then augment, the mediating step that is primarily obstructed in natural infection. Among variable pathogens, that mediator is variability-based humoral evasion (2). To obviate this adaptation, a protective vaccine would need to induce broadly reactive, low-affinity-variance antibodies that target sterically accessible, neutralizing epitopes. Although the latter requirement, based on the multiple-hit hypothesis describing reduced stoichiometric requirements for neutralization of accessible relative to cryptic epitopes, implies variable, accessible epitopes as candidate immunogens, the former suggests evolutionarily constrained epitopes (27). To resolve these competing requirements, an ideal vaccine would elicit neutralizing antibodies that equivalently target variable epitopes by recognizing physiochemically conserved, rather than strain specific, residues.

## Data Availability Statement

The original contributions presented in the study are included in the article/supplementary material, further inquiries can be directed to the corresponding author.

## Author Contributions

The author confirms being the sole contributor of this work and has approved it for publication.

## Conflict of Interest

The author declares that the research was conducted in the absence of any commercial or financial relationships that could be construed as a potential conflict of interest.

## Abbreviations

HCV: hepatitis C virus
HIV: human immunodeficiency virus
AB: antibody
HVR1: hypervariable region 1
nAB: neutralizing antibody
HA: hemagglutinin
BCR: B-cell receptors
bnAB: broadly nAB
AMA1: Ag apical membrane antigen-1.
